# Telpegfilgrastim for chemotherapy-induced neutropenia in patients with non-small cell lung cancer: a multicentre, randomized, phase 3 study

**DOI:** 10.1186/s12885-025-13736-6

**Published:** 2025-03-17

**Authors:** Yuankai Shi, Xinshuai Wang, Zhidong Pei, Huaqiu Shi, Yanjun Zhang, Tienan Yi, Jiazhuan Mei, Yanzhen Guo, Youhong Dong, Tianjiang Ma, Qingyuan Zhang, Xiaojing Jia, Zhengqiu Zhu, Shen Xu, Yanyan Liu, Hongrui Niu, Weimei Jiang, Xiaodong Jiang, Shengyu Zhou, Li Sun

**Affiliations:** 1 Department of Medical Oncology, National Cancer Center/National Clinical Research Center for Cancer/Cancer Hospital, Chinese Academy of Medical Sciences & Peking Union Medical College, Beijing Key Laboratory of Clinical Study On Anticancer Molecular Targeted Drugs, Beijing, China; 2 Department of Oncology, Henan Key Laboratory of Cancer Epigenetics, Cancer Hospital, The First Affiliated Hospital, College of Clinical Medicine, Medical College of Henan University of Science and Technology, Luoyang, China; 3 Department of Oncology, Luoyang Central Hospital Affiliated to Zhengzhou University, Luoyang, China; 4 Department of Oncology, The First Affiliated Hospital of Gannan Medical University, Ganzhou, China; 5 Department of Oncology, Shaanxi Provincial Cancer Hospital, Xi’an, China; 6 Department of Oncology, Xiangyang Central Hospital, Hubei University of Art and Science, Xiangyang, China; 7 Department of Oncology, Zhengzhou People’s Hospital, Zhengzhou, China; 8 Department of Oncology, Xiangyang No. 1 People’s Hospital, Hubei University of Medicine, Xiangyang, China; 9 Department of Oncology, Luohe Central Hospital, Luohe, China; 10 Department of Oncology, Harbin Medical University Cancer Hospital, Harbin, China; 11 Department of Oncology, The Second Hospital of Jilin University, Changchun, China; 12 Department of Oncology, The Affiliated Hospital of Xuzhou Medical University, Xuzhou, China; 13 Department of Medical Oncology, Zhangzhou Municipal Hospital of Fujian Province, Zhangzhou, China; 14 Department of Medical Oncology, The Affiliated Cancer Hospital of Zhengzhou University, Zhengzhou, China; 15 Department of Oncology, The First Affiliated Hospital of Xinxiang Medial University, Xinxiang, China; 16 Department of Oncology, The Second People’s Hospital of Lianyungang, Lianyungang, China; 17 Department of Oncology, The First People’s Hospital of Lianyungang, Lianyungang, China; 18 Xiamen Amoytop Biotech Co., LTD, Xiamen, China

**Keywords:** Recombinant human granulocyte colony stimulating factor, Pegylated recombinant human granulocyte colony stimulating factor, Telpegfilgrastim, Neutropenia, Non-small-cell lung cancer

## Abstract

**Background:**

Chemotherapy-induced neutropenia (CIN) is usually managed by recombinant human granulocyte colony stimulating factor (rhG-CSF) and pegylated rhG-CSF (PEG-rhG-CSF). This study evaluated the efficacy and safety of telpegfilgrastim, a novel Y-shaped PEG-rhG-CSF, for CIN prophylaxis in patients with non-small cell lung cancer (NSCLC).

**Methods:**

This was a multicentre, randomized, open-label, active-controlled non-inferiority study. Patients with NSCLC who received 1–4 chemotherapy cycles of docetaxel plus carboplatin were randomized 1:1:1 to receive telpegfilgrastim 2 mg, 33 µg/kg or control drug (rhG-CSF [Topneuter®] in cycle 1 of chemotherapy, rhG-CSF [Topneuter®] or PEG-rhG-CSF [Xinruibai®] per patients’ choice in cycles 2–4 of chemotherapy). The primary endpoint was duration of grade 4 neutropenia in cycle 1 of chemotherapy. Secondary endpoints included duration of grade 4 neutropenia in cycles 2–4 of chemotherapy, incidence of febrile neutropenia (FN), duration and incidence of ≥ grade 3 neutropenia, dynamic change of absolute neutrophil count from baseline and safety.

**Results:**

From October 16, 2020, to September 1, 2021, 133 patients were randomized to telpegfilgrastim 2 mg (*n* = 44), 33 µg/kg (*n* = 45) and control group (*n* = 44). In cycle 1 of chemotherapy, the mean duration of grade 4 neutropenia in telpegfilgrastim 2 mg, 33 µg/kg groups and control group were 0.02 day, 0.09 day and 0.16 day, respectively. The least square mean differences versus control group were -0.14 day [95% confidence interval [CI]: -0.35, 0.06] for telpegfilgrastim 2 mg group and -0.06 day [95% CI: -0.26, 0.15] for telpegfilgrastim 33 µg/kg group. which met the prespecified non-inferiority margin of 1 day. Incidence of grade 4 neutropenia, incidence of FN and duration of ≥ grade 3 neutropenia in cycles 1–4 of chemotherapy was similar between telpegfilgrastim groups and control group. Telpegfilgrastim was well tolerated, and the incidence of adverse events were comparable with control group.

**Conclusion:**

This study demonstrated that telpegfilgrastim 2 mg or 33 μg/kg was non-inferior to rhG-CSF (Topneuter®) and PEG-rhG-CSF (Xinruibai®) for the management of CIN in patients with NSCLC. In particular, a 2 mg fixed dose of telpegfilgrastim presents a more convenient administration option.

**Trial registration:**

NCT04466137, July 10, 2020.

**Supplementary Information:**

The online version contains supplementary material available at 10.1186/s12885-025-13736-6.

## Background

Lung cancer remains the most common malignancy with the estimated incidence of 2.48 million (12.4%) and 1.82 million (18.7%) deaths worldwide in 2022 [1]. According to 2022 cancer statistics in China, lung cancer is the leading cause of cancer with an incidence of 0.87 million new cases and 0.77 million deaths [2]. Around 85% of lung cancer patients are diagnosed with non-small-cell lung cancer (NSCLC) [3], which has a 5-year survival rate of ~ 20–30% [4]. Conventional platinum-based chemotherapy is the standard-of-care and an effective treatment strategy for NSCLC, but is associated with significant toxicity leading to chemotherapy intolerance and limiting its effectiveness. The major dose-limiting toxicity is chemotherapy-induced neutropenia (CIN). Neutropenia predisposes patients to febrile neutropenia (FN), a potentially life-threatening complication associated with considerable treatment delay, dose reductions and death [5, 6].

Neutropenia and FN can be effectively managed by supportive therapy with recombinant human granulocyte-colony stimulating factor (rhG-CSF) [7, 8]. It involves stimulation, proliferation, differentiation, and survival of neutrophils and thus decreases the incidence of neutropenia and FN in patients receiving myelosuppressive chemotherapy [6]. However, rhG-CSF needs to be administered on a daily basis and has a short plasma half-life of 3–4 h [9]. Pegylated rhG-CSF (PEG-rhG-CSF), a long-acting form of rhG-CSF was developed by covalent binding of a single 20-kDa linear monofunctional monomethoxy-polyethylene glycol (PEG) molecule to the methionine residue at the N-terminus of rhG-CSF [10–12].

PEG-rhG-CSF has increased stability, decreased enzymatic hydrolysis, prolonged plasma half‐life to 30–60 h and decreased fluctuations in blood drug concentrations [5]. Unlike rhG-CSF, PEG-rhG-CSF has the ability to prevent renal clearance and can be cleared through neutrophil-mediated clearance which is dependent on the number of circulating neutrophils. This increases its bioavailability and hence the concentration of PEG-rhG-CSF remains high in serum until the neutrophil counts become normal [13]. It can either be administered as a single dose of 100 µg/kg or as a fixed dose of 6 mg per chemotherapy cycle [5, 10, 13].

Telpegfilgrastim is modified as a 40-kDa Y-shaped PEG-rhG-CSF, developed by Xiamen Amoytop Biotech Co., LTD, Xiamen, China, which extends the half-life to 56.9 ~ 77.4 h [14, 15]. Pegylation with high molecular weight PEGs such as 30-kDa and 40-kDa PEGs, results in significantly prolonged leukocyte proliferation and biological activity, compared to 20-kDa PEGs [16]. In order to prevent the occurrence of CIN in NSCLC patients, telpegfilgrastim is prescribed only once in a chemotherapy cycle at a dosage level lower than that of PEG-rhG-CSF. Therefore, it might add additional value to the patient in terms of dose reductions. Prophylactic administration of telpegfilgrastim once per chemotherapy cycle, maintains a reasonable and effective blood concentration throughout the cycle and prevents the occurrence of neutropenia [17]. Phase 1 study and phase 2 study (data unpublished) have suggested that a dosage of 20 ~ 45 μg/kg telpegfilgrastim is effective in preventing the incidence of CIN and FN. It was generally well tolerated and the drug-related adverse events (AEs) were mainly mild to moderate. Moreover, it does not affect the subsequent treatment and helps the patients to complete the chemotherapy regimen [14, 15]. However, whether telpegfilgrastim can better support the patients with NSCLC undergoing chemotherapy remain to be investigated. Hence, in this phase 3 study, we evaluated the efficacy and safety of telpegfilgrastim in patients with NSCLC for the management of CIN and FN.

## Methods

### Study design

This was a multicentre, randomized, open-label, active drug-controlled phase 3 non-inferiority study conducted at 39 hospitals in China between October 16, 2020 and September 1, 2021. Patients were randomized 1:1:1 using Interactive Web Response System to receive telpegfilgrastim either at a fixed dose of 2 mg or at a dose of 33 μg/kg based on the body weight or control drug (rhG-CSF [Topneuter®] in cycle 1 of chemotherapy, rhG-CSF [Topneuter®] or PEG-rhG-CSF [Xinruibai®] per patients’ choice in cycles 2–4 of chemotherapy). Patients were stratified and randomized based on gender, age (≤ 65 years or > 65 years), and previous chemotherapy status.

The study was approved by each institutional review board/ethics committee of all the participating hospitals and was conducted in accordance with the International Council for Harmonization guidelines on Good Clinical Practice, China’s regulatory requirements and the ethical principles of Declaration of Helsinki. Written informed consents were obtained from all patients before study enrolment. This study was registered at ClinicalTrials.gov, number: NCT04466137.

### Patients

Adult patients aged ≥ 18 years with histopathologically or cytologically confirmed NSCLC who were suitable to receive docetaxel plus carboplatin chemotherapy were included in this study. Patients were considered eligible if they weighed ≥ 45 kg with Karnofsky Performance Status (KPS) ≥ 70, life expectancy ≥ 3 months, white blood cell count ≥ 3.5 × 10^9^/L, platelet count ≥ 100 × 10^9^/L and absolute neutrophil count (ANC) ≥ 1.5 × 10^9^/L. Patients were excluded if they had received chemotherapy in < 2 months before screening, had tumor metastasis in bone marrow, used antibiotics < 72 h before screening, had a history of organ transplantation, bleeding, organ dysfunction, hepatitis, allergy to rhG-CSF, other malignancies, drug/alcohol abuse or were enrolled in any other clinical studies < 3 months before screening.

### Procedure

Patients received combination chemotherapy with intravenous infusion of docetaxel (75 mg/m^2^) and carboplatin (AUC = 5 mg/mL·min, total dose not exceeding 800 mg). A total of 4 cycles of chemotherapy were administered and each chemotherapy cycle were 21 days. Telpegfilgrastim 2 mg fixed dose or 33 µg/kg was subcutaneously injected 48±12 h after the administration of chemotherapy agents in each chemotherapy cycle. In control group, during cycle 1 of chemotherapy, the rhG-CSF [Topneuter®] 5 μg/kg/day was subcutaneously injected 48±12 h after administration of chemotherapy agents, until the ANC dropped to the lowest value and recovered to > 5.0 × 10^9^/L, in no more than 14 days. In cycles 2–4 of chemotherapy, based on the willingness of the patient, a single subcutaneous injection of PEG-rhG-CSF [Xinruibai®] 6 mg or the same regimen of rhG-CSF [Topneuter®] with cycle 1 of chemotherapy was administered.

Blood samples were obtained for routine blood tests and antibody detections. Specifically, routine blood tests were carried out on day 1 (before chemotherapy initiation) and day 3 (prior to study drug administration). Subsequently, daily routine blood tests were implemented until ANC reached its nadir and then increased twice consecutively with ANC ≥ 2.0 × 10^9^/L in the second test. Additionally, a routine blood test was performed on the 21st day thereafter. Serum samples were collected for the detection of anti-drug antibodies (ADAs) and neutralizing antibodies (NABs) at baseline and multiple time points throughout the study period. ADAs were detected using indirect enzyme-linked immunosorbent assay, while NABs were determined by cell proliferation method.

### Endpoints

The primary endpoint was duration of grade 4 neutropenia (defined as ANC < 0.5 × 10^9^/L) in cycle 1 of chemotherapy. The secondary endpoints included duration of grade 4 neutropenia in cycles 2–4 of chemotherapy and incidence of FN, duration and incidence of ≥ grade 3 neutropenia, dynamic change of ANC from baseline in cycles 1–4 of chemotherapy, and safety. FN was defined as ANC < 0.5 × 10^9^/L, or 0.5 × 10^9^/L < ANC < 1.0 × 10^9^/L with prediction to decrease to ANC < 0.5 × 10^9^/L within 48 h, and a single oral temperature > 38.3 °C or a sustained oral temperature ≥ 38.0 °C > 1 h. The ≥ grade 3 neutropenia was defined as ANC < 1.0 × 10^9^/L [18].

Safety assessment included vital signs, clinical laboratory tests, imageological examination, drug exposure dose, and AEs reported at any time during the study. All AEs were graded according to the National Cancer Institute Common Terminology Criteria for Adverse Events version 5.0 [19].

Efficacy and safety were evaluated and compared between telpegfilgrastim treatment groups and control group treated with rhG-CSF in cycle 1 of chemotherapy, whereas in cycles 2–4 of chemotherapy the control group was PEG-rhG-CSF or rhG-CSF based on the willingness of patient.

### Statistical analysis

Assuming the duration of grade 4 neutropenia as 0.5 day with a standard deviation (SD) of 1 day in cycle 1 of chemotherapy in telpegfilgrastim (2 mg fixed dose or 33 µg/kg) and control group, using power analysis software with a predefined non-inferiority margin of 1 day at a power (1-β) of 0.9 and a one-sided test level of 0.025, the study required a minimum sample size of 23 patients in each group. Considering drug exposure and drop-out, a total of 120 patients (40 in each group) were required. Efficacy analyses were performed for patients in the full analysis set (FAS), consisting of all randomized patients who received at least one dose of study drug. If the upper limit of the 95% confidence interval (CI) of the least square (LS) mean difference between the telpegfilgrastim group and the control group is less than 1 day, it is concluded that the test group is non-inferior to the control group. Safety assessment was calculated in the safety set (SS), consisting of all randomized patients who received at least one dose of study drug and at least one safety assessment. Multiple imputation method was used to impute missing data. Differences in duration of grade 4 neutropenia between treatment groups were evaluated using the analysis of variance (ANOVA). Analysis of covariance model was also used to calculate the LS mean and 95% CI. Difference in incidence of neutropenia and FN between different treatment groups were analyzed using Chi-square test. ANOVA were used to assess the differences in duration of ANC recovery between the groups. All tests were two-sided, and the statistical significance was set at *P* < *0*.05. All statistical analyses were performed using SAS version 9.4 (SAS Institute, Cary, NC, USA).

## Results

### Patients disposition

**Fig. 1:**
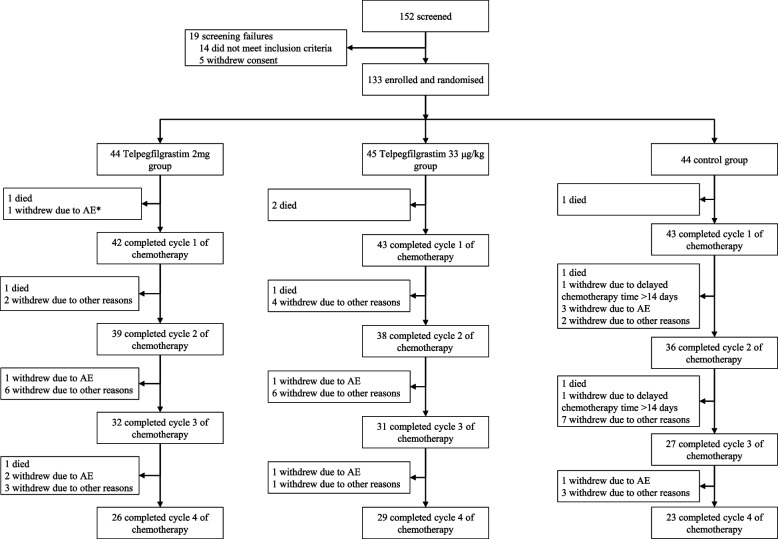
Patients disposition. *This patient developed allergic reactions following chemotherapy before telpegfilgrastim administration and was excluded from the FAS and the SS. Abbreviations: *AE* adverse event, *FAS* full analysis set, *SS* safety set

From October 16, 2020, to September 1, 2021, a total of 152 patients with NSCLC were screened of whom 133 were randomized into telpegfilgrastim groups (2 mg [*n* = 44] or 33 µg/kg [*n* = 45]) and control group (*n* = 44). One patient in telpegfilgrastim 2 mg group developed allergic reactions following chemotherapy before telpegfilgrastim administration and was excluded from the FAS and the SS. Among 132 patients who were included in the FAS and the SS, 128 (97.0%) completed cycle 1 of chemotherapy, 113 (85.6%), 90 (68.2%) and 78 (59.1%) patients completed the cycle 2, cycle 3, and cycle 4 of chemotherapy, respectively. The number of patients completing chemotherapy cycles were similar between the 3 groups (Fig. 1).

The baseline characteristics of patients were presented in Table 1. The mean age of patients was 61.4 (SD, 8.87) years. The 68.2% (90/132) of patients were ≤65 years, 75.8% (100/132) of patients were male, 85.6% (113/132) of patients were advanced stage (stages III or IV), 22.7% (30/132) of patients had previously received chemotherapy, and 7.6% (10/132) of patients had previously received radiotherapy. The mean ANC was 7.3 (SD, 3.77)×10^9^/L. All patients in control group were treated with rhG-CSF in cycle 1 of chemotherapy and PEG-rhG-CSF in cycles 2–4 of chemotherapy.

**Table 1:** Baseline demographic and disease characteristics in the FAS

	**Telpegfilgrastim**		**Control group (*N* = 44)**
	**2 mg group (*N* = 43)**	**33 µg/kg group (*N* = 45)**	
Age (years), mean (SD)	61.0 (8.98)	61.6 (9.54)	61.5 (8.23)
≤ 65 years, n (%)	28 (65.1)	31 (68.9)	31 (70.5)
> 65 years, n (%)	15 (34.9)	14 (31.1)	13 (29.5)
Gender, n (%)			
Male	32 (74.4)	34 (75.6)	34 (77.3)
Female	11 (25.6)	11 (24.4)	10 (22.7)
Nationality, n (%)			
Han	42 (97.7)	45 (100.0)	43 (97.7)
Others	1 (2.3)	0 (0.0)	1 (2.3)
Height (cm), mean (SD)	167.7 (6.73)	165.0 (6.78)	165.9 (8.54)
Weight (kg), mean (SD)	65.4 (11.41)	60.6 (10.95)	61.6 (11.51)
Body surface area (m^2^), mean (SD)	1.7 (0.17)	1.6 (0.17)	1.6 (0.18)
Tumor history – clinical staging, n (%)			
Stage I	3 (7.0)	1 (2.2)	2 (4.5)
Stage II	2 (4.7)	2 (4.4)	3 (6.8)
Stage III	12 (27.9)	11 (24.4)	6 (13.6)
Stage IV	24 (55.8)	30 (66.7)	30 (68.2)
Uncertain	2 (4.7)	1 (2.2)	3 (68)
Previous chemotherapy, n (%)			
Yes	11 (25.6)	7 (15.6)	12 (27.3)
No	32 (74.4)	38 (84.4)	32 (72.7)
Previous radiotherapy, n (%)			
Yes	2 (4.7)	4 (8.9)	4 (9.1)
No	41 (95.3)	41 (91.1)	40 (90.9)
KPS score, mean (SD)	87.2 (6.66)	85.6 (7.55)	85.0 (7.62)
Neutrophils (× 10^9^/L), mean (SD)	7.1 (4.01)	7.8 (3.93)	7.1 (3.37)
Platelets (× 10^9^/L), mean (SD)	267.1 (99.44)	292.4 (97.07)	282.0 (82.28)
Hemoglobin (g/L), mean (SD)	131.3 (15.00)	127.3 (19.45)	125.8 (17.44)

*cm* centimeter, *FAS* Full analysis set, *kg* kilogram, *KPS* Karnofsky Performance Status, *L* Litre, *m* meter, *N* total number, *n* number in respective category, *SD* Standard deviation

### Efficacy

#### Primary endpoint—duration of grade 4 neutropenia in cycle 1 of chemotherapy

The mean duration of grade 4 neutropenia in cycle 1 was 0.02 day and 0.09 day in telpegfilgrastim 2 mg group and telpegfilgrastim 33 µg/kg group, respectively, and it was 0.16 day in control group. The LS mean (95% CI) was 0.06 day (-0.11, 0.22), 0.14 day (-0.03, 0.31) and 0.20 day (0.03, 0.37) in telpegfilgrastim 2 mg group, telpegfilgrastim 33 µg/kg group and control group, respectively. The LS mean difference between telpegfilgrastim 2 mg and telpegfilgrastim 33 µg/kg group with control group was -0.14 day (95% CI: -0.35, 0.06; *P* = *0.175*) and -0.06 day (95% CI: -0.26, 0.15; *P* = *0.571)*, respectively. This supports the non-inferiority of telpegfilgrastim with the control drug (Table 2).

**Table 2:** Summary of efficacy in the FAS

**Efficacy**	**Telpegfilgrastim**		**Control group**	***P*-value telpegfilgrastim 2 mg group vs. control group**	***P*-value telpegfilgrastim 33 μg/kg group vs. control group**
	**2 mg group**	**33 μg/kg group**			
**Duration of grade 4 neutropenia (day), mean ± SD**					
Cycle 1 of chemotherapy	0.02 ± 0.15	0.09 ± 0.47	0.16 ± 0.68	0.175	0.571
Cycle 2 of chemotherapy	0.00 ± 0.00	0.00 ± 0.00	0.03 ± 0.16	0.314	0.320
Cycle 3 of chemotherapy	0.00 ± 0.00	0.07 ± 0.25	0.07 ± 0.37	0.305	0.640
Cycle 4 of chemotherapy	0.00 ± 0.00	0.07 ± 0.37	0.00 ± 0.00	1.000	0.377
**Duration of ≥ grade 3 neutropenia (day), mean ± SD**					
Cycle 1 of chemotherapy	0.02 ± 0.15	0.18 ± 0.68	0.18 ± 0.72	0.312	0.759
Cycle 2 of chemotherapy	0.08 ± 0.47	0.08 ± 0.35	0.08 ± 0.28	0.298	0.638
Cycle 3 of chemotherapy	0.00 ± 0.00	0.16 ± 0.64	0.10 ± 0.56	0.305	0.626
Cycle 4 of chemotherapy	0.11 ± 0.42	0.14 ± 0.58	0.12 ± 0.44	0.953	0.909
**Incidence of grade 4 neutropenia, n/N (%)**					
Cycle 1 of chemotherapy	1/43 (2.3)	2/45 (4.4)	3/44 (6.8)	0.616	0.677
Cycle 2 of chemotherapy	0/40 (0.0)	0/39 (0.0)	1/37 (2.7)	0.481	0.487
Cycle 3 of chemotherapy	0/33 (0.0)	2/31 (6.5)	1/29 (3.4)	0.468	1.000
Cycle 4 of chemotherapy	0/27 (0.0)	1/29 (3.4)	0/25 (0.0)	NA	1.000
**Incidence of ≥ grade 3 neutropenia, n/N (%)**					
Cycle 1 of chemotherapy	1/43 (2.3)	4/45 (8.9)	3/44 (6.8)	0.616	1.000
Cycle 2 of chemotherapy	1/40 (2.5)	2/39 (5.1)	3/37 (8.1)	0.346	0.382
Cycle 3 of chemotherapy	0/33 (0.0)	2/31 (6.5)	1/29 (3.4)	0.468	1.000
Cycle 4 of chemotherapy	2/27 (7.4)	2/29 (6.9)	2/25 (8.0)	1.000	1.000
**Incidence of FN, n/N (%)**					
Cycle 1 of chemotherapy	0/43 (0.0)	1/45 (2.2)	1/44 (2.3)	1.000	1.000
Cycle 2 of chemotherapy	0/40 (0.0)	0/39 (0.0)	2/37 (5.4)	0.228	0.234
Cycle 3 of chemotherapy	0/33 (0.0)	0/31 (0.0)	1/29 (3.4)	0.468	0.483
Cycle 4 of chemotherapy	1/27 (3.7)	0/29 (0.0)	0/25 (0.0)	1.000	NA

*FAS* Full analysis set, *FN* Febrile neutropenia, *N* total number, *NA* Not Available, *n* number in respective category, *SD* Standard deviation

#### Duration and incidence of ≥ grade 3 neutropenia, grade 4 neutropenia

The duration of grade 4 neutropenia in cycles 2–4 was similar among the groups. The duration of ≥ grade 3 neutropenia was also comparable among the groups in all chemotherapy cycles. The incidence of grade 4 neutropenia ranged from 0.0–6.8% in telpegfilgrastim groups and control group. Consistently, the incidence was either numerically similar or lower in telpegfilgrastim 2 mg group compared to 33 µg/kg group and control group. Likewise, incidence of ≥ grade 3 neutropenia was also numerically lowest in telpegfilgrastim 2 mg group except in cycle 4 of chemotherapy. To summarize, duration of grade 4 neutropenia in cycles 2–4 of chemotherapy, duration of ≥ grade 3 neutropenia, incidence of grade 4 and ≥ grade 3 neutropenia did not differ significantly between telpegfilgrastim groups and control group (Table 2).

#### Incidence of FN

In cycle 1 of chemotherapy, the incidence of FN in telpegfilgrastim (2 mg or 33 μg/kg) groups and control (rhG-CSF) group was 0.0%, 2.2% and 2.3% respectively. During cycles 2–4 of chemotherapy, the incidences of FN in telpegfilgrastim 2 mg group were 0.0%, 0.0%, and 3.7% respectively; those in telpegfilgrastim 33 μg/kg group were 0.0%, 0.0%, and 0.0%; and in control (PEG-rhG-CSF) group, they were 5.4%, 3.4%, and 0.0% respectively. No statistically significant differences in the incidence of FN among the groups within each chemotherapy cycle.

#### Changes of ANC compared with baseline

**Fig. 2:**
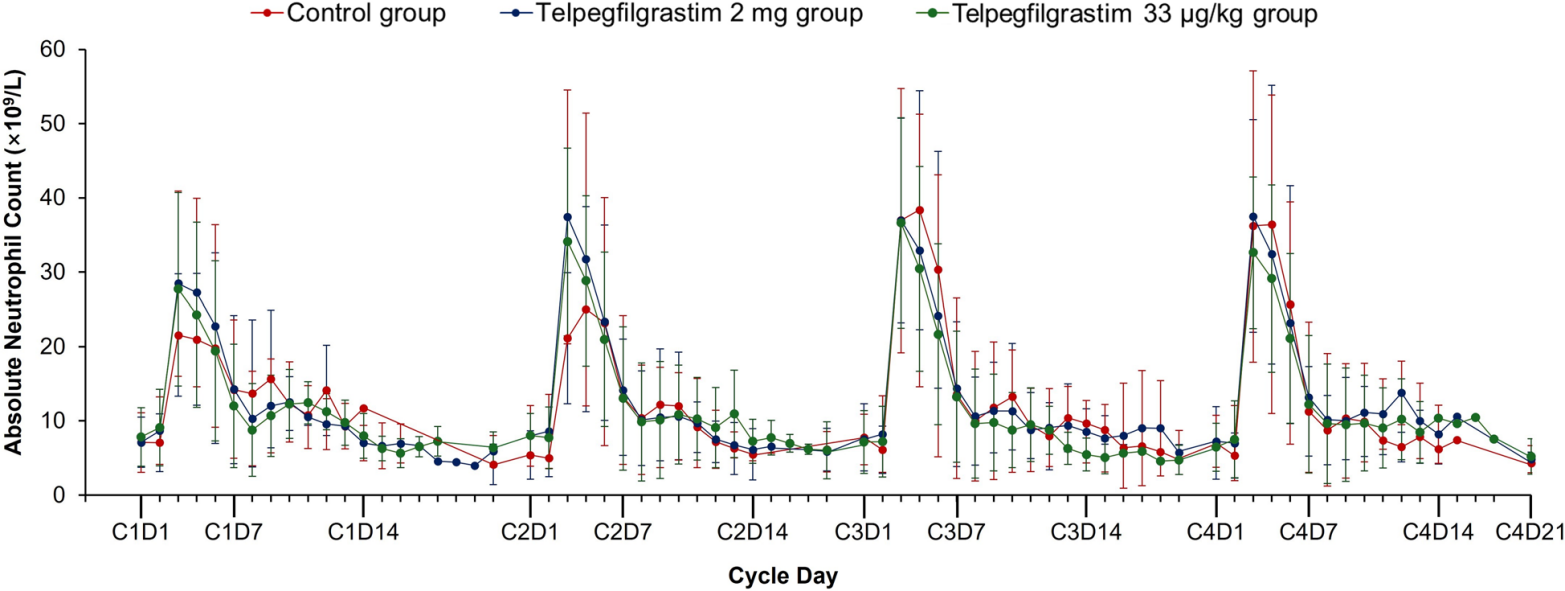
Dynamic changes of ANC in cycles 1–4 of chemotherapy. Data points represent the ANC mean±SD. rhG-CSF was administered in control group during cycle 1 of chemotherapy and PEG-rhG-CSF was administered during cycles 2–4 of chemotherapy. Abbreviations: *ANC* absolute neutrophil count, *C* cycle, *D* day, *rhG-CSF* recombinant human granulocyte colony stimulating factor, *PEG-rhG-CSF* pegylated recombinant human granulocyte colony stimulating factor, *SD* Standard deviation

After a single subcutaneous injection of telpegfilgrastim (2 mg or 33 ug/kg), the peak ANC value appeared on the 4th day after chemotherapy. In control group, the peak ANC occurred on the 4th day of chemotherapy after rhG-CSF administration in cycle 1 of chemotherapy and on the 5th day of chemotherapy after administration of PEG-rhG-CSF in cycles 2–4 of chemotherapy. The ANC peak achieved was wider with PEG-rhG-CSF administration than the telpegfilgrastim group during cycles 3–4 of chemotherapy (Fig. 2).

The average time for ANC returns to ≥ 2.0 × 10^9^/L in cycle 1 of chemotherapy was numerically lowest in telpegfilgrastim 2 mg group at 0.07 (± 0.34) day compared to 0.24 (± 0.61) day in telpegfilgrastim 33 µg/kg group and 0.14 (± 0.46) day in control group, though the difference between groups was not statistically significant (*P* = *0.236).* In cycles 2–4 of chemotherapy, the mean time for ANC recovers to ≥ 2.0 × 10^9^/L still less than 1 day.

#### Antibiotic medications and chemotherapy completion

The antibiotic usage rates of telpegfilgrastim 2 mg group, telpegfilgrastim 33 µg/kg group and control group during study period were 20.9% (9/43), 17.8% (8/45) and 22.7% (10/44), respectively (*P* > 0.05). The proportion of patients who completed the expected chemotherapy in telpegfilgrastim 2 mg group, telpegfilgrastim 33 µg/kg group and control group were all 100% in cycles 1–3 of chemotherapy, 96.3%, 100% and 100% in cycle 4 of chemotherapy, respectively. The overall exposure and compliance of chemotherapy drugs was found to be good.

#### Subgroup analysis of efficacy in cycle 1 of chemotherapy

To determine whether the stratification factors impacted the assessment of efficacy endpoints, a predefined subgroup analysis according to age (≤ 65 years vs. > 65 years), gender (male vs. female), and prior chemotherapy status (yes vs. no) was performed for cycle 1 of chemotherapy. The results revealed no significant differences in primary and secondary endpoints in the above-mentioned subgroups. (Table S1).

### Safety

The incidence of AEs was similar between two telpegfilgrastim groups and control group throughout the study period. The incidence, severity and type of AEs related to the study drug were similar to rhG-CSF and PEG-rhG-CSF. AEs were 98.5% (130/132) in the SS. Treatment emergent adverse events (TEAEs) were 97.0% (128/132) while ≥ grade 3 TEAEs were 39.4% (52/132) in the SS. Treatment related adverse events (TRAEs) were 34.8% (46/132) in the SS, whereas the incidence of TEAEs related to chemotherapy drugs was 94.7% (125/132) in the SS. The incidence of ≥ grade 3 TRAEs was 2.3% (3/132) (Table 3). The incidence of TRAEs were similar in telpegfilgratim groups (2 mg or 33 µg/kg) and control group, which were 39.5% (17/43), 33.3% (15/45), and 31.8% (14/44), respectively.

**Table 3:** AEs in the SS (*N* = 132)

**AEs, n (%)**	**Telpegfilgrastim**		**Control group (*N* = 44)**
	**2 mg group (*N* = 43)**	**33 µg/kg group (*N* = 45)**	
All AEs	43 (100)	44 (97.8)	43 (97.7)
TEAEs	42 (97.7)	44 (97.8)	42 (95.5)
≥ Grade 3 TEAEs	17 (39.5)	14 (31.1)	21 (47.7)
TRAEs	17 (39.5)	15 (33.3)	14 (31.8)
≥ Grade 3 TRAEs	2 (4.7)	0	1 (2.3)
TEAEs associated with chemotherapy drugs	42 (97.7)	42 (93.3)	41 (93.2)
TEAEs leading to study withdrawal	6 (14.0)	5 (11.1)	8 (18.2)
TRAEs leading to study withdrawal	1 (2.3)	0	1 (2.3)
Serious TEAEs	6 (14.0)	6 (13.3)	7 (15.9)
Serious TRAEs	0	0	1 (2.3)
TRAEs occuring in ≥ 5% of patients			
Asthenia	5(11.6)	1(2.2)	5(11.4)
Fever	1(2.3)	3(6.7)	1(2.3)
Platelet count decreased	4(9.3)	0	1(2.3)
Platelet count increased	0	3(6.7)	3(3.4)
Nausea	5(11.6)	1(2.2)	3(6.8)
Decreased appetite	1(2.3)	2(4.4)	5(11.4)
Bone pain	1(2.3)	0	3(6.8)
Anemia	2(4.7)	3(6.7)	1(2.3)

*AEs* Adverse events, *N* total number, *n* number in respective category, *TEAEs* Treatment emergent adverse events, *TRAEs* Treatment related adverse events, *SS* Safety set

## Immunogenicity

ADA-positive at baseline in telpegfilgrastim (2 mg or 33 µg/kg) groups were 9.3% (4/43) and 6.7% (3/45) respectively, while in control group it was 4.5% (2/44). After multiple cycles of chemotherapy, in baseline ADA negative patients, the incidence was 0 (0/32), 2.9% (1/34) for telpegfilgrastim 2 mg group and telpegfilgrastim 33 µg/kg group respectively, and 15.6% (5/32) in control group. In baseline ADA positive patients, the incidence was 0 (0/4), 100% (3/3), 100% (1/1) in telpegfilgrastim 2 mg group, telpegfilgrastim 33 µg/kg group, and control group, respectively. At baseline, the incidence of NABs was 4.7% (2/43), 0 (0/45) and 2.3% (1/44) in telpegfilgrastim 2 mg group, telpegfilgrastim 33 µg/kg group, and control group, respectively. However, no new NABs were detected during multiple chemotherapy cycles (Table S2).

## Discussion

The present study evaluated the efficacy and safety of telpegfilgrastim in preventing neutropenia during multiple cycles of chemotherapy in patients with NSCLC. The results of this study demonstrated that the efficacy and safety of telpegfilgrastim was non-inferior to rhG-CSF (Topneuter®) and PEG-rhG-CSF (Xinruibai®).

Telpegfilgrastim was reported to reduce the incidence of CIN and FN in cancer patients receiving chemotherapy [14, 15]. In this study, the mean duration of grade 4 neutropenia in telpegfilgrastim groups (2 mg: 0.02 day; 33 µg/kg: 0.09 day) showed comparable results with rhG-CSF (0.16 day) in cycle 1 of chemotherapy. It was similar in cycles 2–4 of chemotherapy compared to PEG-rhG-CSF. The result thereby establishes equivalent efficacy of telpegfilgrastim with control drugs for prophylaxis of grade 4 neutropenia. A phase 3 study has previously established equivalent efficacy between different dose regimens (6 mg or 100 µg/kg) of mecapegfilgrastim and rhG-CSF with respect to the mean duration of grade 4 neutropenia in Chinese patients with NSCLC [6]. Similar non-inferior results of PEG-rhG-CSF 6 mg compared with rhG-CSF 5 µg/kg/day have been observed in breast cancer or other solid tumors [11, 20, 21]. In a phase 3 study, the mean duration of grade 4 neutropenia was shown to be 1.8 days in PEG-rhG-CSF group compared with 1.6 days in rhG-CSF group in cycle 1 of chemotherapy. It was 1.1 days, 1.1 days and 1.0 day in PEG-rhG-CSF group compared to 0.9 day, 0.9 day and 1.0 day in rhG-CSF group in cycles 2–4 of chemotherapy, respectively [13]. Therefore, the results from this study and previous studies in NSCLC and other solid tumors studies suggest that telpegfilgrastim can be used as an effective alternative to rhG-CSF and PEG-rhG-CSF for the prophylaxis of chemotherapy-induced grade 4 neutropenia in chemotherapy-treated patients with NSCLC.

The duration of ≥ grade 3 neutropenia in this study in telpegfilgrastim groups (2 mg: 0.02 day; 33 µg/kg; 0.18 day) and in control group who received rhG-CSF (0.18 day) had no significant difference between the study groups in cycle 1 of chemotherapy. Notably, the dose of telpegfilgrastim used in this study (2 mg fixed dose or 33 µg/kg) was 3-times lower than that of other PEG-rhG-CSF (6 mg or 100 µg/kg) but was still effective in reducing the durations of ≥ grade 3 neutropenia. In this study, the incidence of grade 4 neutropenia and ≥ grade 3 neutropenia during cycle 1 of chemotherapy in telpegfilgrastim 2 mg group, telpegfilgrastim 33 µg/kg group, and control group was 2.3% and 2.3%, 4.4% and 8.9%, 6.8% and 6.8%, respectively. The results of this study indicated that telpegfilgrastim (2 mg or 33 µg/kg) was effective for the prophylaxis of chemotherapy induced grade 4 and ≥ grade 3 neutropenia in patients with NSCLC.

PEG-rhG-CSF has been shown to be effective in management of FN and neutropenia-related complications in patients with solid tumors receiving myelosuppressive chemotherapy [22]. In an earlier randomized clinical study on breast cancer patients receiving chemotherapy, a significant reduction was reported in the incidence of FN with PEG-rhG-CSF compared to placebo (1% vs. 17%) [23]. Similar reductions were noted in colorectal cancer patients in the incidence of FN (2% vs. 8%) compared to placebo [24]. Therapeutic use of PEG-rhG-CSF resulted in significantly shorter mean recovery time of FN (*P* = *0.038)* and grade 3/4 neutropenia (*P* = *0.000*) compared to rhG-CSF in Chinese patients with breast cancer [21]. Prophylactic PEG-rhG-CSF regimens were shown to be effective in reducing FN risk in a variety of non-myeloid malignancies with acceptable safety profile [25]. American Society of Clinical Oncology [26] and the European Organization for Research and Treatment of Cancer [27] reported a FN incidence of 26% in patients with NSCLC receiving docetaxel plus carboplatin chemotherapy. FN incidence ranging between 4%-20% was noted in other studies in patients with lung cancer [28–31]. With telpegfilgrastim intervention in this study, FN occurred only 1 patient in telpegfilgrastim group and 4 patients in control group who received PEG-rhG-CSF in cycles 2–4 of chemotherapy.

ANC levels recovered within 1 day in telpegfilgrastim groups and control group in all 4 chemotherapy cycles. The time taken to achieve ANC peak in telpegfilgrastim groups (4th day) is same as control group who received rhG-CSF (4th day) in cycle 1 of chemotherapy. In cycles 2–4 of chemotherapy, the time was shorter in telpegfilgrastim groups (4th day) than control group who received PEG-rhG-CSF (5th day). However, there was no statistical difference between telpegfilgrastim groups and control group. The results were similar to the previously published results of pegfilgrastim and mecapegfilgrastim [6, 32]. In cycles 3–4 of chemotherapy, ANC peak appeared to be wider after administration of PEG-rhG-CSF. ANC reached a higher level on the 4th day of chemotherapy cycle but the peak value lags behind and appeared on the 5th day, suggesting that ANC stays at a higher level for a longer time after administration of PEG-rhG-CSF. It should be noted that the recommended dose of PEG-rhG-CSF was 6 mg, which was 3 times higher than the dosage of telpegfilgrastim (2 mg). Maintaining high level of ANC for a long time might result in excessive bone marrow stimulation which can lead to insufficient bone marrow stimulation in subsequent chemotherapy cycle and may also cause severe AEs such as splenomegaly, spleen rupture, and lung infiltration. As no lag in the peak values of neutrophils, telpegfilgrastim can be effective for the prophylaxis of chemotherapy induced neutropenia in NSCLC patients.

Telpegfilgrastim demonstrated favourable safety profile and good tolerability. The incidence of TRAEs in telpegfilgrastim group was comparable with that in control group. The main TRAEs in this study were asthenia, fever, nausea, decreased appetite, bone pain and anemia. Musculoskeletal pain, fever, chills, body aches, flu symptoms, shortness of breath and allergic reactions were the AEs reported to be associated with PEG-rhG-CSF [32]. Incidence of pain was noted in 35% of patients with breast cancer treated with 6 mg PEG-rhG-CSF [33]. It was reported to be 15.7% in patients with NSCLC treated with 3 mg PEG-rhG-CSF [32] and 17.9% in Chinese patients with various solid tumors receiving PEG-rhG-CSF [11]. In this study, the incidence of bone pain was lower in telpegfilgrastim groups and control group. The incidence of bone pain was 2.3% in telpegfilgrastim 2 mg and 0.0% in telpegfilgrastim 33ug/kg group, while it was 6.8% in control group. The incidence of ADAs was 0% and 2.9% in telpegfilgrastim 2 mg group and telpegfilgrastim 33 µg/kg group, respectively, while it was 15.6% in control group. Furthermore, No new NABs after 4 cycles of chemotherapy, suggesting that telpegfilgrastim does not trigger the formation of NABs during treatment, which is encouraging for long-term usage.

The major limitation of this study is only included NSCLC patients treated with docetaxel plus carboplatin. Though the study found no significant differences in subgroup analyzes of primary and secondary endpoints based on age and previous chemotherapy, it must be noted that other NSCLC chemotherapy regimens should also be considered for further investigation.

## Conclusion

This study demonstrated that telpegfilgrastim 2 mg or 33 μg/kg was non-inferior to rhG-CSF (Topneuter®) and PEG-rhG-CSF (Xinruibai®) for the management of CIN in patients with NSCLC. In particular, a 2 mg fixed dose of telpegfilgrastim presents a more convenient administration option.

## Supplementary Information

Supplementary Material 1.

## Data Availability

The data that support the findings of this study are available from the corresponding author upon reasonable request.

## Abbreviations

CIN: Chemotherapy-induced neutropenia
rhG-CSF: Recombinant human granulocyte colony stimulating factor
PEG-rhG-CSF: Pegylated recombinant human granulocyte colony stimulating factor
NSCLC: Non-small-cell lung cancer
FN: Febrile neutropenia
ANC: Absolute neutrophil count
AEs: Adverse events
KPS: Karnofsky Performance Status
SD: Standard deviation
FAS: Full analysis set
CI: Confidence interval
LS: Least square
SS: Safety set
ANOVA: Analysis of variance
TEAEs: Treatment emergent adverse events
TRAEs: Treatment related adverse events
