# Supplementary material for: Telpegfilgrastim for chemotherapy-induced neutropenia in patients with non-small cell lung cancer: a multicentre, randomized, phase 3 study
Source: BMC Cancer. 2025 Mar 17;25:490. doi: 10.1186/s12885-025-13736-6 (PMC11916207; doi:10.1186/s12885-025-13736-6)
Supplement: Supplementary file 1 — Supplementary Material 1. [file 12885_2025_13736_MOESM1_ESM.docx]

***Supplementary File***

**Telpegfilgrastim for Chemotherapy-Induced Neutropenia in Patients with Non-Small Cell Lung Cancer: A Multicentre, Randomized, Phase 3 Study**

Yuankai Shi^1*^, Xinshuai Wang^2^, Zhidong Pei^3^, Huaqiu Shi^4^, Yanjun Zhang^5^, Tienan Yi^6^, Jiazhuan Mei^7^, Yanzhen Guo^2^, Youhong Dong^8^, Tianjiang Ma^9^, Qingyuan Zhang^10^, Xiaojing Jia^11^, Zhengqiu Zhu^12^, Shen Xu^13^, Yanyan Liu^14^, Hongrui Niu^15^, Weimei Jiang^16^, Xiaodong Jiang^17^, Shengyu Zhou^1^，Li Sun^18^

^1^Department of Medical Oncology, National Cancer Center/National Clinical Research Center for Cancer/Cancer Hospital, Chinese Academy of Medical Sciences & Peking Union Medical College, Beijing Key Laboratory of Clinical Study on Anticancer Molecular Targeted Drugs, Beijing, China

^2^Department of Oncology,Henan Key Laboratory of Cancer Epigenetics, Cancer Hospital, The First Affiliated Hospital, College of Clinical Medicine, Medical College of Henan University of Science and Technology, Luoyang, China

^3^Department of Oncology, Luoyang Central Hospital Affiliated to Zhengzhou University, Luoyang, China

^4^Department of Oncology, the First Affiliated Hospital of Gannan Medical University, Ganzhou, China

^5^Department of Oncology, Shaanxi Provincial Cancer Hospital, Xi'an, China

^6^Department of Oncology, Xiangyang Central Hospital, Hubei University of Art and Science, Xiangyang, China

^7^Department of Oncology, Zhengzhou People’s Hospital, Zhengzhou, China

^8^Department of Oncology, Xiangyang No. 1 People's Hospital, Hubei University of Medicine, Xiangyang, China

^9^Department of Oncology, Luohe Central Hospital, Luohe, China

^10^Department of Oncology, Harbin Medical University Cancer Hospital, Harbin, China

^11^Department of Oncology,The second hospital of Jilin University, Changchun，China

^12^Department of Oncology, The Affiliated Hospital of Xuzhou Medical University, Xuzhou, China

^13^Department of Medical Oncology, Zhangzhou Municipal Hospital of Fujian Province, Zhangzhou, China

^14^Department of Medical Oncology, The Affiliated Cancer Hospital of Zhengzhou University, Zhengzhou, China

^15^Department of Oncology, The First Affiliated Hospital of Xinxiang Medial University, Xinxiang, China

^16^Department of Oncology, The Second People’s Hospital of Lianyungang, Lianyungang, China

^17^Department of Oncology, The First People’s Hospital of Lianyungang, Lianyungang, China

^18^Xiamen Amoytop Biotech Co., LTD, Xiamen, China

***Corresponding author:**

Yuankai Shi,

Department of Medical Oncology,

National Cancer Center/National Clinical Research Center for Cancer/Cancer Hospital,

Chinese Academy of Medical Sciences & Peking Union Medical College,

Beijing Key Laboratory of Clinical Study on Anticancer Molecular Targeted Drugs,

Beijing, China. Email: [syuankai@cicams.ac.cn](mailto:syuankai@cicams.ac.cn)

**Table S1** Subgroup analysis of efficacy based on age, gender and prior chemotherapy status during cycle 1 of chemotherapy in the FAS (N=132)

| Efficacy | Telpegfilgrastim | | Control group  (N=44) |
| --- | --- | --- | --- |
|  | **2 mg group (N=43)** | **33 μg/kg group (N=45)** |  |
| Age | | | |
| Incidence of grade 4 neutropenia,, n (%) |  |  |  |
| ≤ 65 years | 1 (3.6) | 0 (0.0) | 2 (6.5) |
| > 65 years | 0 (0.0) | 2 (14.3) | 1 (7.7) |
| Duration of grade 4 neutropenia, (day), mean±SD |  |  |  |
| ≤ 65 years | 0.04±0.19 | 0.0±0.0 | 0.19±0.79 |
| > 65 years | 0.00±0.00 | 0.29±0.83 | 0.08±0.28 |
| Incidence of ≥ grade 3 neutropenia,, n (%) |  |  |  |
| ≤ 65 years | 1 (3.6) | 1 (3.2) | 2 (6.5) |
| > 65 years | 0 (0.0) | 3 (21.4) | 1 (7.7) |
| Duration of ≥ grade 3 neutropenia, (day), mean±SD |  |  |  |
| ≤ 65 years | 0.04±0.19 | 0.03±0.18 | 0.19±0.79 |
| > 65 years | 0.00±0.00 | 0.50±1.16 | 0.15±0.55 |
| Incidence of FN, n (%) |  |  |  |
| ≤ 65 years | 0 (0.0) | 0 (0.0) | 1 (3.2) |
| > 65 years | 0 (0.0) | 1 (7.1) | 0 (0.0) |
| Gender | | | |
| Incidence of grade 4 neutropenia,, n (%) |  |  |  |
| Male | 1 (3.1) | 1 (2.9) | 3 (8.8) |
| Female | 0 (0.0) | 1 (9.1) | 0 (0.0) |
| Duration of grade 4 neutropenia, (day), mean±SD |  |  |  |
| Male | 0.03±0.18 | 0.08±0.51 | 0.21±0.77 |
| Female | 0.00±0.00 | 0.09±0.30 | 0.0±0.00 |
| Incidence of ≥ grade 3 neutropenia,, n (%) |  |  |  |
| Male | 1 (3.1) | 2 (5.9) | 3 (8.8) |
| Female | 0 (0.0) | 2 (18.2) | 0 (0.0) |
| Duration of ≥ grade 3 neutropenia, (day), mean±SD |  |  |  |
| Male | 0.03±0.18 | 0.15±0.70 | 0.24±0.82 |
| Female | 0.00±0.00 | 0.27±0.65 | 0.0±0.00 |
| Incidence of FN, n (%) |  |  |  |
| Male | 0 (0.0) | 1 (2.9) | 1 (2.9) |
| Female | 0 (0.0) | 0 (0.0) | 0 (0.0) |
| Prior chemotherapy status | | | |
| Incidence of grade 4 neutropenia,, n (%) |  |  |  |
| Prior chemotherapy | 0 (0.0) | 0 (0.0) | 2 (16.7) |
| No prior chemotherapy | 1 (3.1) | 2 (5.3) | 1 (3.1) |
| Duration of grade 4 neutropenia, (day), mean±SD |  |  |  |
| Prior chemotherapy | 0.00±0.00 | 0.00±0.00 | 0.50±1.24 |
| No prior chemotherapy | 0.03±0.18 | 0.11±0.51 | 0.03±0.18 |
| Incidence of ≥ grade 3 neutropenia,, n (%) |  |  |  |
| Prior chemotherapy | 0 (0.0) | 1 (14.3) | 2 (16.7) |
| No prior chemotherapy | 1 (3.1) | 3 (7.9) | 1 (3.1) |
| Duration of ≥ grade 3 neutropenia, (day),  mean±SD | |  |  |
| Prior chemotherapy | 0.00±0.00 | 0.14±0.38 | 0.50±1.24 |
| No prior chemotherapy | 0.03±0.18 | 0.18±0.73 | 0.06±0.35 |
| Incidence of FN, n (%) |  |  |  |
| Prior chemotherapy | 0 (0.0) | 0 (0.0) | 1 (8.3) |
| No prior chemotherapy | 0 (0.0) | 1 (2.6) | 0 (0.0) |
| *FAS* full analysis set, *FN* febrile neutropenia, *N* total number of patients, *n* number of patients in the respective category, *SD* standard deviation. | | | |

**Table S2. ADAs and NABs in telpegfilgrastim groups (2 mg or 33 μg/kg) and control group**

| **Immunogenicity** | **Telpegfilgrastim** | | **Control group**  **(N=44)** |
| --- | --- | --- | --- |
|  | **2 mg group**  **(N=43)** | **33 μg/kg group**  **(N=45)** |  |
| **Baseline, N** | **43** | **45** | **44** |
| ADA (+), n(%) | 4 (9.3) | 3 (6.7) | 2 (4.5) |
| NAB (+), n(%) | 2 (4.7) | 0 | 1 (2.3) |
| **Multi-cycle administration, N** | **36** | **37** | **33** |
| Baseline ADA (-),N | 32 | 34 | 32 |
| ADA (+), n(%) | 0 | 1 (2.9) | 5 (15.6) |
| NAB (+), n(%) | 0 | 0 | 0 |
| Baseline ADA (+),N | 4 | 3 | 1 |
| ADA (+), n(%) | 0 | 3 (100) | 1 (100) |
| NAB (+), n(%) | 0 | 0 | 0 |
| *ADA* anti-drug antibody, *N* total number of patients, *NAB* neutralizing antibody, *n* total number of patients in specific group. | | | |
